# Poor guideline adherence in the initiation of antidepressant treatment in children and adolescents in the Netherlands: choice of antidepressant and dose

**DOI:** 10.1007/s00787-016-0836-3

**Published:** 2016-03-17

**Authors:** Ymkje Anna de Vries, Peter de Jonge, Luuk Kalverdijk, Jens H. J. Bos, Catharina C. M. Schuiling-Veninga, Eelko Hak

**Affiliations:** 1 Department of Psychiatry, Interdisciplinary Center Psychopathology and Emotion Regulation, University of Groningen, University Medical Center Groningen, Groningen, The Netherlands; 2 Department of Psychiatry, University Medical Center Groningen, Groningen, The Netherlands; 3 Unit PharmacoEpidemiology and PharmacoEconomics (PE2), Department of Pharmacy, University of Groningen, Groningen, The Netherlands

**Keywords:** Antidepressants, Guidelines, Children and adolescents, Dosing, Prescription trends

## Abstract

The Dutch guideline for the treatment of depression in young people recommends initiating antidepressant treatment with fluoxetine, as the evidence for its efficacy is strongest and the risk of suicidality may be lower than with other antidepressants. Furthermore, low starting doses are recommended. We aimed to determine whether antidepressant prescriptions are in accord with guidelines. A cohort of young people aged between 6 and 17 at the time of antidepressant initiation was selected from IABD, a Dutch pharmacy prescription database. The percentage of prescriptions for each antidepressant was determined. Starting and maintenance doses were determined and compared with recommendations for citalopram, fluoxetine, fluvoxamine, and sertraline. During the study period, 2942 patients initiated antidepressant treatment. The proportion of these young people who were prescribed fluoxetine increased from 10.1 % in 1994–2003 to 19.7 % in 2010–2014. However, the most commonly prescribed antidepressants were paroxetine in 1994–2003 and citalopram in 2004–2014. The median starting and maintenance doses were ≤0.5 DDD/day for tricyclic antidepressants and 0.5–1 DDD/day for SSRIs and other antidepressants. Starting doses were guideline-concordant 58 % of the time for children, 31 % for preteens, and 16 % for teens. Sixty percent of teens were prescribed an adult starting dose. In conclusion, guideline adherence was poor. Physicians preferred citalopram over fluoxetine, in contrast to the recommendations. Furthermore, although children were prescribed a low starting dose relatively frequently, teens were often prescribed an adult starting dose. These results suggest that dedicated effort may be necessary to improve guideline adherence.

**Electronic supplementary material:**

The online version of this article (doi:10.1007/s00787-016-0836-3) contains supplementary material, which is available to authorized users.

## Introduction

Practice guidelines in the Netherlands [1] and internationally [e.g., 2] recommend that medication should only be prescribed to children and adolescents suffering from (moderate to) severe depression. These guidelines also recommend that pharmacotherapy should be initiated with fluoxetine, with sertraline or citalopram used in case of non-response to fluoxetine. Other antidepressants, such as mirtazapine, venlafaxine, and tricyclic antidepressants, are not recommended. In addition, treatment should be initiated with a low starting dose (a quarter to a half of the adult starting dose) [3, 4].

Second-generation antidepressants may be moderately effective for depression in children and adolescents [5], but they have also been associated with an increased risk of suicidal ideation and behavior [6]. In 2004, the United States Food and Drug Administration issued a black box warning on antidepressants to emphasize the risk of suicidality in young people. There is some evidence to suggest that the risk may vary by antidepressant, with fluoxetine showing less of an increased risk than many other second-generation antidepressants [7]. The risk of suicidality may also be dose-related, with young people prescribed higher-than-modal starting doses of antidepressants showing an increased risk of suicidal behavior compared to those prescribed the modal dose [8]. Further epidemiological evidence in adults also suggests that lower-than-modal doses may be associated with decreased risk, although confounding by indication cannot be excluded. This study also found that risk was particularly increased within the first 3 months after starting an antidepressant [9].

Although prescription trends in children have been examined extensively [e.g., 10–17], most studies have not examined specifically the first prescription of an antidepressant and only one study, to our knowledge, has examined whether appropriate dosages are used [18]. This study found that antidepressant treatment in young people in the USA was more commonly initiated with a low dose after the black box warning was issued in 2004, although low doses were still only prescribed in a minority of cases. In the Netherlands, health insurance reimbursements show that citalopram is the most commonly prescribed antidepressant to young people [19], which suggests that the guidelines may not be followed; however, as this includes all antidepressant prescriptions and citalopram is recommended as a second antidepressant, the evidence is not yet conclusive. To our knowledge, no evidence is currently available regarding antidepressant dosing in the Netherlands. In the current study, we therefore aimed to answer the following questions: first, do physicians initiate antidepressant treatment in young people with fluoxetine? Second, what are the usual starting and maintenance doses of antidepressants in young people, and are these in accord with the guidelines?

## Methods

### Data source

Prescription data were obtained from the IADB database, which contains information on prescriptions filled in community pharmacies in the Netherlands between 1994 and 2014 [20]. The population included in the database in any given year is currently approximately 600,000. Patients are included in the database the first time they fill a prescription in one of the participating pharmacies. The database includes information about the patient (gender, date of birth) and the prescription [fill date, Anatomical Therapeutical Chemical (ATC) code, number of tablets, daily dose (in number of tablets), and the total number of defined daily doses (DDDs) in the prescription]. A DDD is defined as the assumed average maintenance dose for a drug used for its main indication in adults [21]. All outpatient prescriptions are included in the database, but inpatient prescriptions and over-the-counter medications are not.

### Patient selection

From the IADB database, a cohort of young patients initiating treatment with an antidepressant was selected. We included tricyclic antidepressants (ATC-code N06AA), selective serotonin reuptake inhibitors (SSRIs, ATC-code N06AB), and other antidepressants (N06AX). Monoamine oxidase inhibitors (selective and non-selective MAOIs, ATC-codes N06AF and N06AG) were not included, as it is unlikely that a MAOI would be prescribed as the first antidepressant. Patients were included in the cohort if they were between 6 and 17 years of age (inclusive), had been included in the database for at least 6 months at the time of first prescription of an antidepressant, and had not previously received a prescription for a different antidepressant. Patients aged between six and nine were categorized as children; patients aged between ten and thirteen as preteens; and patients aged between fourteen and seventeen as teens. For patients who had multiple episodes of antidepressant treatment during the study period, we only considered data from the first eligible treatment episode.

We excluded patients who were likely prescribed an antidepressant for non-psychiatric indications, specifically bed-wetting and pain. Patients starting on amitriptyline or imipramine who also received a prescription for desmopressin (H01BA02), the first-line treatment for bed-wetting, at any time during the study period were excluded (238 patients). We also excluded patients who received two or more prescriptions for pain-related medication within 6 months prior to initiation of a tricyclic antidepressant (111 patients). Pain-related medication was defined as any medication with ATC-code M01 (anti-inflammatory and anti-rheumatic drugs), N02 (analgesics), N03AX12 (gabapentin), and N03AX16 (pregabalin). One patient received a first prescription for two different antidepressants on the same day and was also excluded.

### Data analysis: type of antidepressant

We split the data into three time periods: 1994 through 2003, 2004 through 2009, and 2010 through 2014. These time periods were chosen based upon major events: in 2004, knowledge of a possible link between antidepressants and suicidality in children became widespread, while in December 2009, the youth addendum to the Dutch Multidisciplinary Guideline for Depression was published.

We determined which antidepressant was first prescribed to each patient and calculated the percentage of patients who were prescribed fluoxetine as their first antidepressant. Possible moderators were examined by stratifying the data based upon prescriber (general practitioner (GP) or specialist) and age group (child, preteen, or teen).

### Data analysis: starting and maintenance dose

We determined the starting dose of antidepressants (in DDD/day) for each patient. A conversion of DDDs to the equivalent dose in milligrams for the ten most commonly prescribed antidepressants is provided in Table 1. The dose was calculated as the total number of DDDs divided by the total number of days in the first prescription. The number of days was calculated by dividing the total number of units (pills) by the number of pills to take daily. For patients who received multiple prescriptions for the same antidepressant on the same day, we took the prescription with the lowest daily dose. Additionally, for prescriptions for the highly concentrated liquid formulations of citalopram and escitalopram, we divided the daily dose by 20 (as 1 drop of solution is approximately equivalent to 0.05 ml) before calculating the DDD/day. We excluded patients with missing daily doses [4 (0.1 %) patients] or with unrealistically low [0 DDD/day, 256 (8.7 %) patients] or high [>3 DDD/day, 6 (0.2 %) patients] doses, as these are likely to reflect data entry errors (in particular, entering 0 as the number of units per day).

**Table 1:** Dose in milligrams equivalent to 1 defined daily dose (DDD) of the ten most commonly prescribed antidepressants

Drug	1 DDD-equivalent (mg)
Imipramine	100
Clomipramine	100
Amitriptyline	75
Fluoxetine	20
Citalopram	20
Paroxetine	20
Sertraline	50
Fluvoxamine	100
Mirtazapine	30
Venlafaxine	100

The maintenance dose of antidepressants was determined in a similar fashion. Maintenance was defined as a period in which at least two prescriptions with the same dose were filled, containing a minimum of 60 days’ supply. Prescriptions were required to be overlapping, i.e., the number of days in the first prescription must be sufficient to cover the fill date of the subsequent prescription, after adding 25 % to the number of days to account for possible non-compliance. For patients who had multiple maintenance periods, we selected the period with the longest duration and the highest dose (if multiple periods had the same duration). Missing doses were set to 0. We excluded patients with unrealistically low [0 DDD/day, 8 (0.1 %) patients] or high maintenance doses (>4 DDD/day, 0 patients). Fifty-three percent of all patients had at least one maintenance period with a realistic dose.

The distribution of starting doses and maintenance doses was determined for each antidepressant. For the SSRIs fluoxetine, citalopram, sertraline, and fluvoxamine, the distribution was compared to the Dutch dosing guidelines for children [3, 4]. These guidelines recommend a starting dose of 5 mg (0.25 DDD) for fluoxetine and citalopram, and 25 mg (0.25 DDD) for fluvoxamine. For sertraline, the guidelines both recommend 25 mg (0.5 DDD) for young children, but one guideline recommends a higher dose of 50 mg (1 DDD) for adolescents aged 13 and older [3]. We chose to compare the distribution to the latter, more lenient guideline. Subsequently the data were stratified by prescriber and by age group to examine the possible moderating influence of these variables. We also examined how many patients were prescribed a starting dose of fluoxetine of 10 mg or less, as recommended in international guidelines (e.g., United Kingdom [2]).

## Results

### Demographics

A total of 2942 patients were prescribed a first antidepressant during the study period and met inclusion criteria: 1194 in 1994–2003, 815 in 2004–2009, and 933 in 2010–2014. Of these patients, 1739 (59 %) were female, 1188 (40 %) were male, and for 15 (1 %) information about sex was missing. The average age of the sample at initiation was 14.2 years. Three-hundred and eleven (11 %) patients were children, 573 (19 %) were preteens, and 2058 (70 %) were teens. The majority of patients (62 %) received their first prescription for an antidepressant from their GP in 1994–2003, but by 2010–2014 69 % of patients received their first prescription from a specialist.

### First antidepressant

**Fig. 1:**
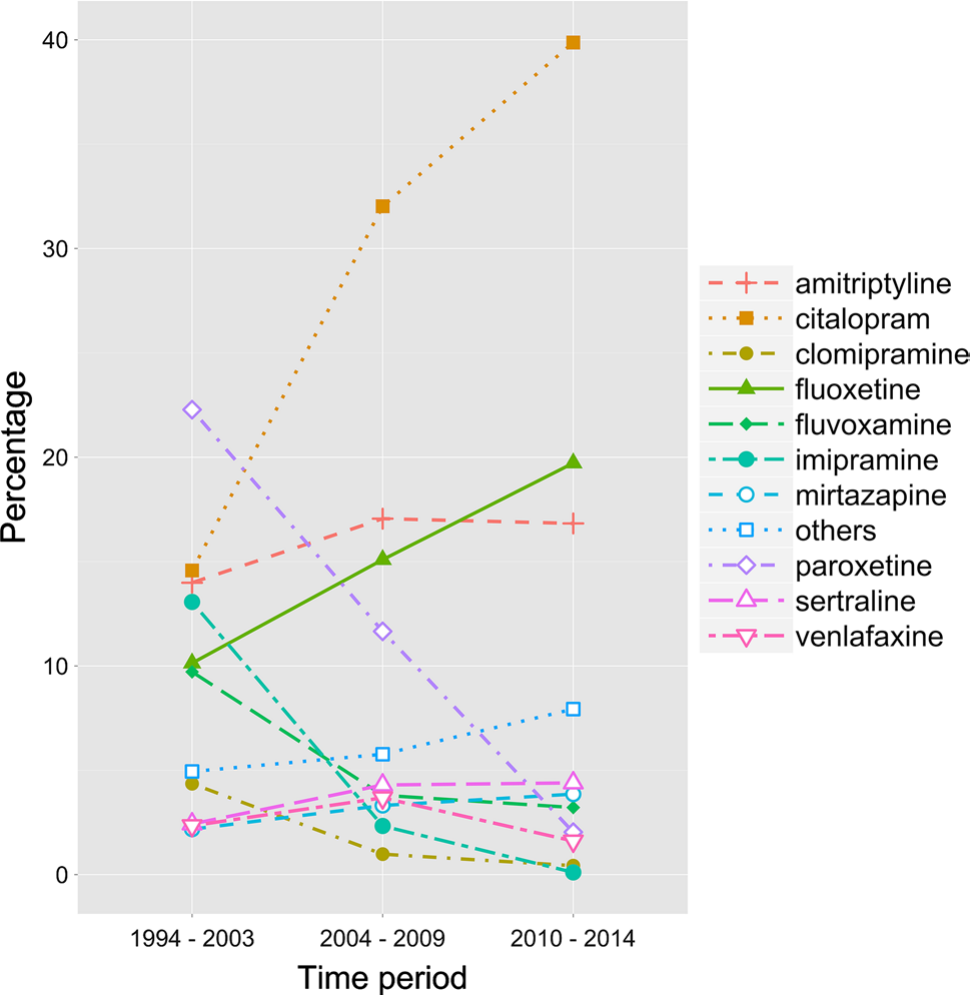
Percentage of young people prescribed one of the ten most commonly prescribed antidepressants in each time period. Prescriptions for all other antidepressants are combined into the category “others”

Of all young people initiating treatment with an antidepressant, the proportion prescribed fluoxetine increased during the study period from 10.1 % in 1994–2003 to 19.7 % in 2010–2014 (Fig. 1), but fluoxetine was never the most commonly prescribed antidepressant. Instead, antidepressant treatment was most commonly initiated with paroxetine in 1994–2003 and with citalopram from 2004 onward. A full listing of all antidepressants is provided in supplemental Table 1.

**Fig. 2:**
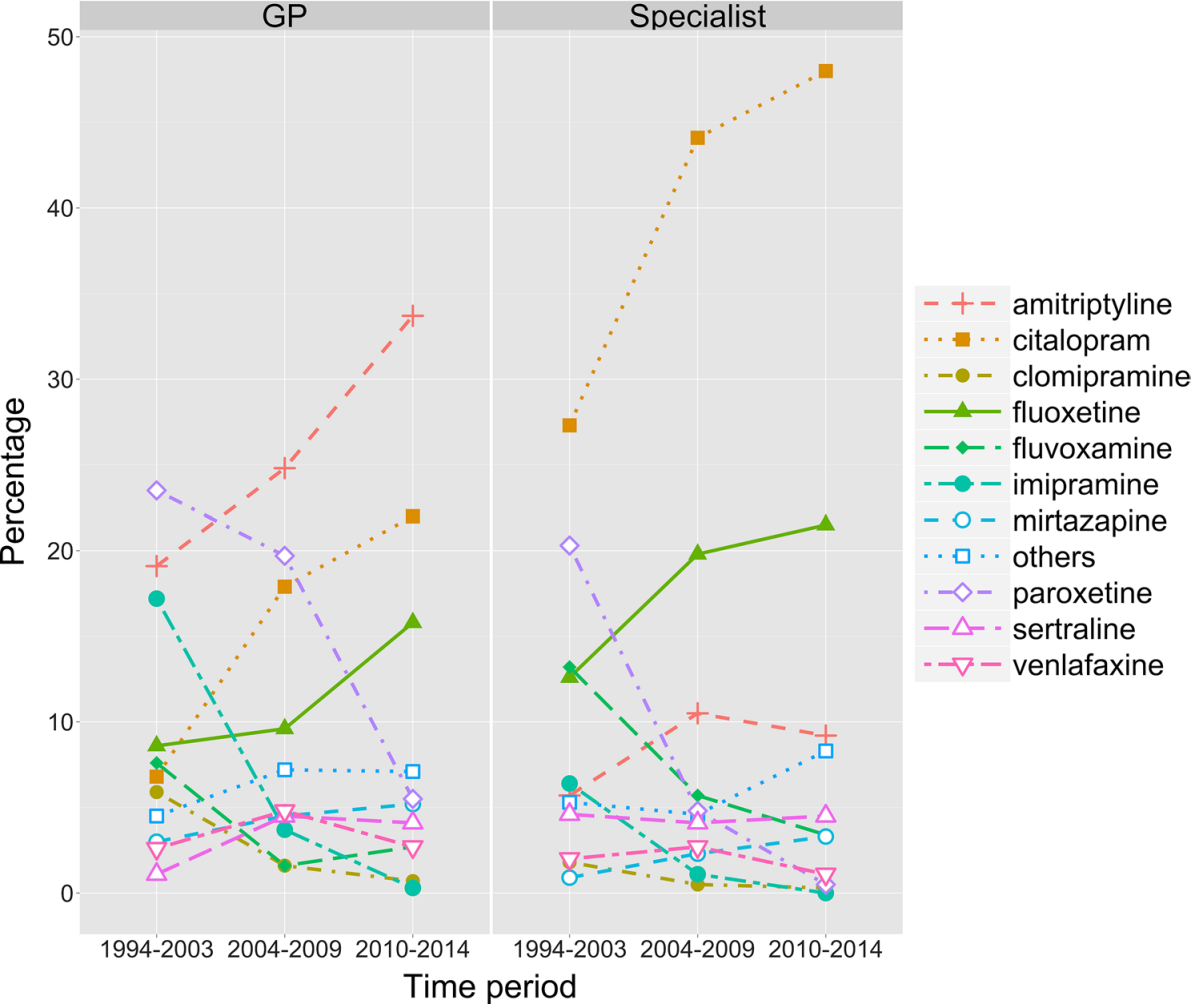
Percentage of young people prescribed one of the ten most commonly prescribed antidepressants in each time period, stratified by prescriber. Prescriptions for all other antidepressants are combined into the category “others”

Stratification by prescriber (Fig. 2) showed that specialists were slightly more likely to prescribe fluoxetine than GPs at each time point. In 2010–2014, GPs initiated treatment with fluoxetine in 15.8 % of cases, while specialists did so in 21.5 % of all cases. Specialists most commonly initiated treatment with citalopram, while GPs most commonly initiated treatment with amitriptyline. Both GPs and specialists showed a steep decrease in the use of paroxetine, although this decrease occurred earlier for specialists than for GPs.

**Fig. 3:**
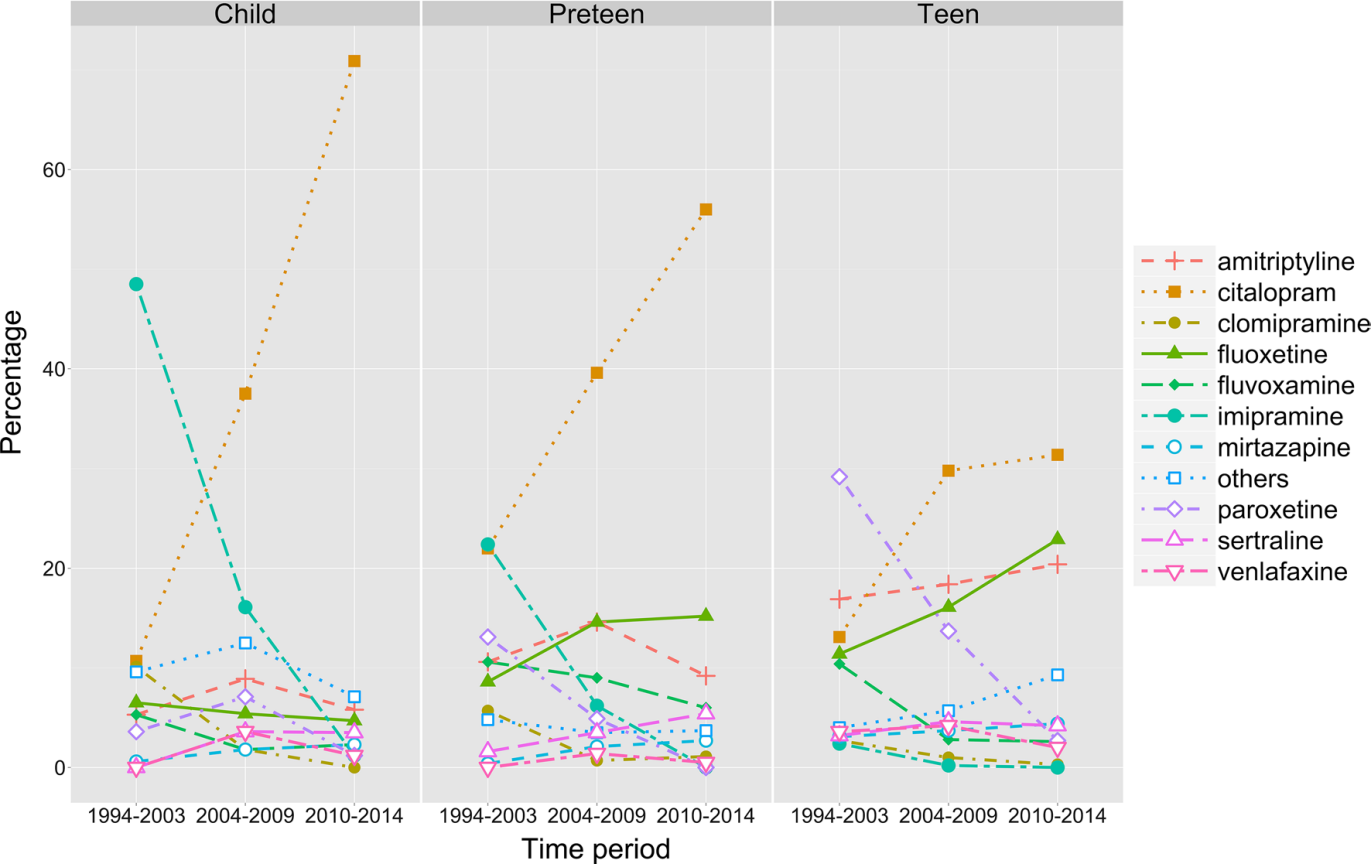
Percentage of young people prescribed one of the ten most commonly prescribed antidepressants in each time period, stratified by age group. Prescriptions for all other antidepressants are combined into the category “others”

Stratification by age group showed low rates of antidepressant initiation with fluoxetine in each age group (Fig. 3). Children were prescribed fluoxetine least frequently (<6.5 % throughout the study period). Preteens were prescribed fluoxetine in 8.6 % of cases in 1994–2003, increasing to 15.2 % in 2010–2014. Teens were prescribed fluoxetine relatively frequently, at 11.4 % in 1994–2003 and 22.9 % in 2010–2014. Citalopram was the most commonly prescribed antidepressant in all age groups by 2010–2014. In particular, children and preteens received citalopram in 70.9 and 56.0 % of cases, respectively, in 2010–2014.

### Starting doses

**Fig. 4:**
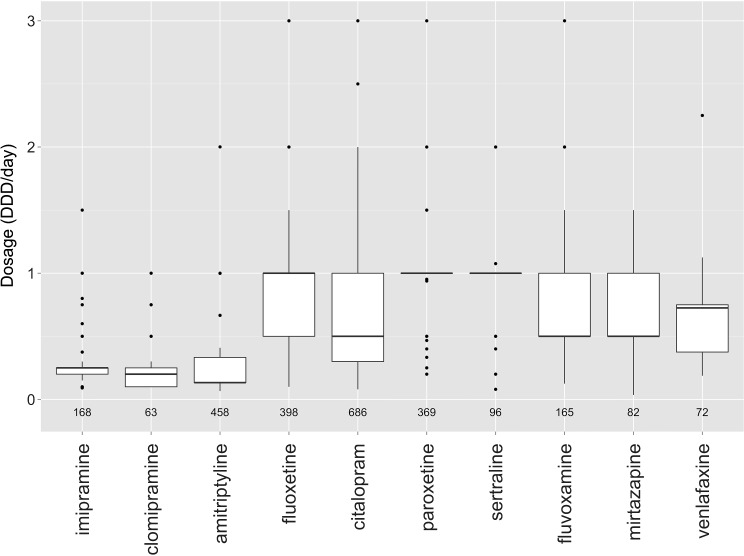
Distribution of starting doses for the ten most commonly prescribed antidepressants. The starting dose is expressed as the number of DDDs per day, where 1 DDD is defined as the assumed average maintenance dose for a drug used in its main indication in adults. The numbers below the *boxplots* indicate the number of prescriptions for that antidepressant

The distribution of starting doses for the 10 most commonly prescribed antidepressants is shown in Fig. 4. A full listing is given in supplemental Table 2. The median starting dose for tricyclic antidepressants was quite low, at around 0.1–0.3 DDD/day. For SSRIs, the median starting dose was 1 DDD/day for fluoxetine, paroxetine, and sertraline, while it was 0.5 DDD/day for citalopram and fluvoxamine. Mirtazapine and venlafaxine had median starting doses of 0.5 and 0.75 DDD/day, respectively. A minority of first prescriptions (22.2 %) were according to guidelines: 6.5 % for fluoxetine, 24.9 % for citalopram, 86.5 % for sertraline, and 14.5 % for fluvoxamine.

Median starting doses were similar throughout the study period for most antidepressants, but decreased for citalopram, paroxetine, mirtazapine, and venlafaxine. For citalopram, the median starting dose decreased from 1 DDD/day in 1994–2003 to 0.4 DDD/day in 2010–2014; for paroxetine and mirtazapine, the median starting dose decreased from 1 DDD/day in 1994–2003 to 0.5 DDD/day in 2010–2014; and for venlafaxine, the median starting dose decreased from 0.75 DDD/day in 1994–2003 to 0.375 DDD/day in 2010–2014. Guideline adherence also improved somewhat within the study period for citalopram and fluvoxamine, with adherence rates being 44.3 % for citalopram and 28.6 % for fluvoxamine in 2010–2014.

Stratification by prescriber showed few differences between prescribers, although specialists prescribed some SSRIs in slightly lower doses than GPs. Stratification by age showed that children tended to be prescribed lower doses (≤0.5 DDD/day), particularly for the SSRIs. Preteens received slightly higher doses: 0.5 DDD/day for all SSRIs except sertraline (1 DDD/day). Teens received the highest doses, at 1 DDD/day for all SSRIs except fluvoxamine (0.5 DDD/day). Table 2 shows the percentage of first prescriptions according to guidelines for fluoxetine, citalopram, sertraline, and fluvoxamine, stratified by age group. Children were reasonably likely to be prescribed according to guidelines (58 % across all four antidepressants), although 10 % of children were prescribed an adult starting dose. On the other hand, very few teens (16 %) were prescribed according to guidelines, while 60 % of teens were prescribed an adult starting dose. For fluoxetine specifically, 33 % of children, 16 % of preteens and 3 % of teens received a guideline-compliant dose (≤0.25 DDD/day). The corresponding percentages for a fluoxetine dose of ≤0.5 DDD/day (10 mg) were 67 % for children, 58 % for preteens, and 30 % for teens.

**Table 2:** Comparison of antidepressant starting dose with guidelines and with adult doses

	Guideline-compliant dose			Adult dose (≥1 DDD/day)		
	Child (*N* = 115)	Preteen (*N* = 295)	Teen (*N* = 961)	Child (*N* = 115)	Preteen (*N* = 295)	Teen (*N* = 961)
	*n* (%)	*n* (%)	*n* (%)	*n* (%)	*n* (%)	*n* (%)
Fluoxetine	5 (33.3)	10 (16.1)	11 (3.4)	2 (13.3)	25 (40.3)	222 (69.2)
Citalopram	49 (66.2)	62 (36.3)	60 (13.6)	6 (8.1)	32 (18.7)	231 (52.4)
Sertraline	3 (60.0)	13 (72.2)	67 (91.8)	2 (40.0)	10 (55.6)	62 (84.9)
Fluvoxamine	4 (36.4)	6 (13.0)	14 (13.0)	0 (0.0)	8 (17.4)	50 (46.3)
Total	61 (58.1)	91 (30.8)	152 (15.8)	10 (9.5)	75 (25.3)	565 (59.9)

*n* indicates the number of prescriptions that were at or below the guideline dose, while *N* indicates the total number of prescriptions for all four antidepressants in that age group

### Maintenance doses

Maintenance doses were similar to starting doses (supplemental Table 3). The median maintenance dose for tricyclic antidepressants was around 0.2–0.3 DDD/day. For SSRIs, the median maintenance dose was 1 DDD/day. For the other antidepressants, median maintenance doses were 0.5 DDD/day for mirtazapine and 0.75 DDD/day for venlafaxine. Maintenance doses were nearly always according to guidelines for fluoxetine (98 %), citalopram (96 %), sertraline (91 %), and fluvoxamine (93 %).

Among those who had a valid starting dose as well as a valid maintenance dose, 60 % remained at their starting dose, while 35 % titrated up to a higher dose and 5 % titrated down. These percentages were similar across prescribers and age groups; however, they did vary according to the antidepressant prescribed. Of the 10 most commonly prescribed antidepressants, up-titration was more likely for citalopram (46 %), sertraline (42 %), and venlafaxine (47 %), while it was less likely for imipramine (25 %), amitriptyline (17 %), paroxetine (24 %), and mirtazapine (21 %).

## Discussion

### Principal findings

Physicians initiated pharmacotherapy with fluoxetine less than 20 % of the time, even after publication of the guidelines for youth in 2009. The percentage of first prescriptions for paroxetine decreased sharply after 2003, a trend which is most likely due to its particularly prominent association with suicidality in young people. Our results suggest that prescriptions for paroxetine were not replaced with fluoxetine, as the guidelines suggest, but with citalopram, which became the most popular antidepressant by 2004–2009. Although citalopram is effective for depression in adults [22], it has not been shown to be effective in children and adolescents, in contrast to fluoxetine [5], which is the only second-generation antidepressant registered for the treatment of depression in young people in the Netherlands and many other countries. Antidepressants may also be prescribed for anxiety, particularly in younger children, but no randomized placebo-controlled trial of citalopram for that purpose appears to have been conducted in children and adolescents, although fluoxetine has been found effective [23, 24]. Among the SSRIs, citalopram has also been most strongly associated with QT interval prolongation (particularly at higher doses), which may increase the risk for torsade de pointes and sudden cardiac death [25, 26] and which may be an additional safety-related reason, apart from treatment-emergent suicidality, to prefer fluoxetine as a first-line treatment.

The starting dose of antidepressants was generally higher than recommended. In particular, teens were usually prescribed an adult starting dose and were only rarely prescribed according to guidelines. Young children were prescribed according to the guidelines much more frequently (58 %), but 10 % of children were actually prescribed the adult starting dose, which is two to four times higher than the recommended dose. Few differences between prescribers were apparent, although specialists prescribed some SSRIs in slightly lower starting doses than GPs. This may be due to the slightly lower mean age of children receiving SSRIs from specialists compared to GPs.

Sertraline and citalopram were more likely to be prescribed according to the guidelines than other antidepressants. For sertraline, this is likely because the recommended starting dose is higher than that of other antidepressants, especially for older children. If we had used the stricter guideline rather than the more lenient guideline, adherence would have been markedly lower (23 % overall). For citalopram, the higher adherence to guidelines may be due to the availability of a liquid solution for citalopram, which facilitates low starting doses. In contrast, for fluoxetine, the tablet with the lowest dose currently available in the Netherlands contains 20 mg (1 DDD), which makes it difficult to provide the recommended dose of 5 mg. Although liquid fluoxetine was previously available, it is not currently on the Dutch market. The difficulty of providing low doses of fluoxetine may be one reason for physicians’ preference for citalopram.

Several positive findings were also apparent. While GPs prescribed the majority of antidepressants in 1994–2003, prescriptions shifted to specialists over time, as recommended by guidelines. We also found that the starting doses of some antidepressants, particularly citalopram, decreased over the study period, suggesting increasing awareness among physicians of the importance of low starting doses in young people. This finding agrees with a previous study in the USA showing increased prescription of low doses after the FDA warning in 2004 [18]. Finally, maintenance doses were nearly always in agreement with the guidelines; where they were not, this was usually because the dose was lower than recommended. In general, maintenance doses were very similar to starting doses. Up-titration from a low starting dose is recommended in the guidelines, but titration occurred in a minority of cases, probably because the starting dose was already within the maintenance range. Up-titration was more likely for second-generation antidepressants like citalopram and venlafaxine, for which a relatively low starting dose was also more likely.

The number of young people initiating antidepressant treatment decreased in the early 2000s, followed by a return to the level of 2001. Such a trend was also found in countries like the UK [13], but only to a slight extent or not at all in other countries, such as Canada [27] or Denmark [28]. The decrease in antidepressant initiation in young people was likely related to media coverage of the potential for treatment-emergent suicidality with antidepressant treatment [15], but this effect appears to have been transient.

### Improving guideline adherence

Adherence to guidelines is often poor [29], and physicians’ prescription choices are influenced by a multitude of other factors besides guidelines and continuing medical education. These influences may include the mass media (which may have been especially important with regard to the reduction in prescriptions for paroxetine) [15] and promotion by pharmaceutical companies [30]. A large body of research has examined barriers and facilitators to the implementation of guidelines in clinical practice [31–33]. Adherence is more likely when recommendations are specific and concrete rather than vague, when few additional resources are required for implementation, and when the evidence is strong and straightforward [33, 34]. While the recommendation to initiate antidepressant treatment in children with fluoxetine is highly specific and does not require any additional resources, the evidence base for the use of fluoxetine in young people is relatively limited, although stronger than that for other antidepressants [5], which may affect physicians’ confidence in the recommendation. Dedicated effort, for example implementation interventions [35], may be needed to improve adherence to guidelines. A variety of interventions have been found to increase guideline adherence, including provision of educational materials, audit and feedback, and reminders, but effects are modest [36]. Educational meetings, which are a common form of continuing medical education, also have small effects on improving guideline adherence [37]. A better understanding of the reasons behind physicians’ preference for citalopram may help clarify how guideline adherence could be improved.

### Strengths and limitations

This study has several strengths. First, use of a general population prescription database excludes the possibility of recall bias and selection bias. Another important strength is that we specifically examined first prescriptions, in contrast to many previous studies. Furthermore, we included a long time period of 21 years, which allowed us to examine time trends and the possible influence of major events, such as the recognition of a link between antidepressants and suicidality in young people in 2003–2004. This long time period also included very recent data (up to and including 2014).

Some limitations must also be acknowledged. An important limitation is that we did not have information about the indication for a prescription. As the guideline recommending fluoxetine is a guideline for the treatment of depression in young people, it may not apply to all prescriptions included herein. In particular, amitriptyline was frequently prescribed in children and adolescents (approximately 15 % of all prescriptions), even though tricyclic antidepressants are not recommended for the treatment of depression. Although we attempted to remove prescriptions for bed-wetting and pain, the remaining patients may still have been treated with amitriptyline for complaints other than depression. A study among Dutch GPs suggested that SSRIs were usually prescribed for depression or anxiety, but tricyclics were often prescribed for bed-wetting, hyperactivity, tension headache or non-specific disease, and only rarely for depression [38]. Consequently, without information on the indication for amitriptyline prescriptions, it is difficult to determine whether these prescriptions were appropriate (although bed-wetting is the only approved indication for children and adolescents in the Netherlands). However, as the majority of SSRI prescriptions to children and adolescents are for the purpose of treating depression [38], this limitation does not invalidate our finding that citalopram is preferred over fluoxetine, in contrast to the guideline. Furthermore, low starting doses are important regardless of the indication and might even be of greater importance if antidepressants are prescribed for the treatment of anxiety, the most probable alternative indication for SSRIs, given the potential for increased anxiety early in treatment [39].

A second limitation of our study is that inpatient prescriptions are not included in the database. Consequently, some ‘first prescriptions’ may actually have been repeat prescriptions after treatment initiation during hospitalization. However, only 3–4 % of all children who are treated in specialist mental health care are hospitalized in a year [40].

## Conclusions

The guidelines on the treatment of depression in youth recommend fluoxetine as the treatment of choice. However, Dutch physicians appear to prefer citalopram over fluoxetine, even though citalopram has not been studied extensively and meta-analysis does not support its superiority over placebo in a pediatric population [5]. This is in contrast to findings from other countries, such as the United Kingdom, where antidepressant treatment in young people was most commonly initiated with fluoxetine (although citalopram has gained in popularity) [13]. Given that UK guidelines are similar to Dutch guidelines, this suggests that factors other than guidelines are likely to be the strongest driving forces behind (changes in) prescription patterns.

Furthermore, physicians tend to prescribe adult starting doses to older children. Although teens may weigh as much as adults, the possibility of a dose–response relationship with suicidality [8, 9] suggests that caution should be exercised, even for older children. The same may also apply to young adults, for whom antidepressants have also been shown to increase the risk of suicidality [41]. Although starting doses were adjusted for children and preteens, they were still frequently higher than recommended. Maintenance doses, on the other hand, were usually within the recommended range.

Taken together, these findings show that adherence to guidelines for antidepressant initiation in children and adolescents is poor. In light of the limited evidence for the efficacy of some antidepressants and the potential for treatment-emergent suicidality, physicians should be made aware of the importance of guideline adherence and cautious dosing of antidepressants in children and adolescents.

## Electronic supplementary material

Below is the link to the electronic supplementary material.

Supplementary material 1 (DOCX 25 kb)
