# Supplementary material for: Poor guideline adherence in the initiation of antidepressant treatment in children and adolescents in the Netherlands: choice of antidepressant and dose
Source: Eur Child Adolesc Psychiatry. 2016 Mar 17;25(11):1161–70. doi: 10.1007/s00787-016-0836-3 (PMC5083767; doi:10.1007/s00787-016-0836-3)
Supplement: Supplementary file 1 — Supplementary material 1 (DOCX 25 kb) [file 787_2016_836_MOESM1_ESM.docx]

| **Antidepressant** | **ATC** | **Incident users (n (%))** | | |
| --- | --- | --- | --- | --- |
|  |  | **1994 – 2003** | **2004 – 2009** | **2010 - 2014** |
| Desipramine | N06AA01 | 9 (0.8) | 1 (0.1) | 0 (0) |
| Imipramine | N06AA02 | 156 (13.1) | 19 (2.3) | 1 (0.1) |
| Clomipramine | N06AA04 | 52 (4.4) | 8 (1.0) | 4 (0.4) |
| Amitriptyline | N06AA09 | 167 (14.0) | 139 (17.1) | 157 (16.8) |
| Nortriptyline | N06AA10 | 5 (0.4) | 5 (0.6) | 8 (0.9) |
| Doxepin | N06AA12 | 5 (0.4) | 0 (0) | 0 (0) |
| Dosulepin | N06AA16 | 1 (0.1) | 0 (0) | 0 (0) |
| Maprotiline | N06AA21 | 3 (0.3) | 0 (0) | 1 (0.1) |
| Fluoxetine | N06AB03 | 121 (10.1) | 123 (15.1) | 184 (19.7) |
| Citalopram | N06AB04 | 174 (14.6) | 261 (32.0) | 372 (39.9) |
| Paroxetine | N06AB05 | 266 (22.3) | 95 (11.7) | 19 (2.0) |
| Sertraline | N06AB06 | 29 (2.4) | 35 (4.3) | 41 (4.4) |
| Fluvoxamine | N06AB08 | 116 (9.7) | 31 (3.8) | 30 (3.2) |
| Escitalopram | N06AB10 | 0 (0) | 11 (1.3) | 28 (3.0) |
| Oxitriptan | N06AX01 | 1 (0.1) | 0 (0) | 0 (0) |
| Mianserin | N06AX03 | 1 (0.1) | 0 (0) | 0 (0) |
| Trazodone | N06AX05 | 2 (0.2) | 7 (0.9) | 5 (0.5) |
| Nefazodone | N06AX06 | 4 (0.3) | 0 (0) | 0 (0) |
| Mirtazapine | N06AX11 | 26 (2.2) | 27 (3.3) | 36 (3.9) |
| Bupropion | N06AX12 | 3 (0.3) | 11 (1.3) | 13 (1.4) |
| Venlafaxine | N06AX16 | 28 (2.3) | 30 (3.7) | 15 (1.6) |
| Duloxetine | N06AX21 | 0 (0) | 3 (0.4) | 7 (0.8) |
| Agomelatine | N06AX22 | 0 (0) | 0 (0) | 5 (0.5) |
| St. John’s wort | N06AX25 | 25 (2.1) | 9 (1.1) | 7 (0.8) |
| **Total** |  | **1194 (100)** | **815 (100)** | **933 (100)** |

Supplemental table 1: Number (and percentage) of young people initiating treatment with each antidepressant (prescribed at least once during the study period).

| **Antidepressant** | **ATC** | **N** | **Median [IQR]** |
| --- | --- | --- | --- |
| Desipramine | N06AA01 | 8 | 0.50 [0.38 – 0.75] |
| Imipramine | N06AA02 | 168 | 0.25 [0.20 – 0.25] |
| Clomipramine | N06AA04 | 63 | 0.20 [0.10 – 0.25] |
| Amitriptyline | N06AA09 | 458 | 0.13 [0.13 – 0.33] |
| Nortriptyline | N06AA10 | 15 | 0.13 [0.13 – 0.67] |
| Doxepin | N06AA12 | 4 | 0.25 [0.18 – 0.33] |
| Dosulepin | N06AA16 | 1 | 0.17 |
| Maprotiline | N06AA21 | 3 | 0.25 [0.25 – 0.25] |
| Fluoxetine | N06AB03 | 398 | 1.00 [0.50 - 1.00] |
| Citalopram | N06AB04 | 686 | 0.50 [0.30 – 1.00] |
| Paroxetine | N06AB05 | 369 | 1.00 [1.00 – 1.00] |
| Sertraline | N06AB06 | 96 | 1.00 [1.00 – 1.00] |
| Fluvoxamine | N06AB08 | 165 | 0.50 [0.50 – 1.00] |
| Escitalopram | N06AB10 | 35 | 1.00 [0.50 – 1.00] |
| Oxitriptan | N06AX01 | 0 | - |
| Mianserin | N06AX03 | 1 | 0.5 |
| Trazodone | N06AX05 | 13 | 0.33 [0.33 – 0.33] |
| Nefazodone | N06AX06 | 4 | 0.50 [0.50 – 0.63] |
| Mirtazapine | N06AX11 | 82 | 0.50 [0.50 – 1.00] |
| Bupropion | N06AX12 | 23 | 0.50 [0.50 – 0.50] |
| Venlafaxine | N06AX16 | 72 | 0.72 [0.375 – 0.75] |
| Duloxetine | N06AX21 | 8 | 0.50 [0.50 – 0.75] |
| Agomelatine | N06AX22 | 4 | 1.00 [1.00 – 1.50] |
| St. John’s wort | N06AX25 | 0 | - |
| **Total** |  | **2676** | **0.50 [0.25 – 1.00]** |

Supplemental table 2: Median [IQR] of the starting dose for all included antidepressants. Note: no defined daily dose has been set for St. John’s wort (N06AX25).

| **Antidepressant** | **ATC** | **N** | **Median [IQR]** |
| --- | --- | --- | --- |
| Desipramine | N06AA01 | 5 | 0.75 [0.75 – 0.75] |
| Imipramine | N06AA02 | 46 | 0.25 [0.25 – 0.50] |
| Clomipramine | N06AA04 | 26 | 0.25 [0.20 – 0.50] |
| Amitriptyline | N06AA09 | 123 | 0.20 [0.13 – 0.33] |
| Nortriptyline | N06AA10 | 11 | 0.67 [0.40 – 0.83] |
| Doxepin | N06AA12 | 1 | 0.5 |
| Dosulepin | N06AA16 | 0 | - |
| Maprotiline | N06AA21 | 0 | - |
| Fluoxetine | N06AB03 | 290 | 1.00 [1.00 – 1.00] |
| Citalopram | N06AB04 | 487 | 1.00 [0.50 – 1.00] |
| Paroxetine | N06AB05 | 239 | 1.00 [1.00 – 1.00] |
| Sertraline | N06AB06 | 76 | 1.00 [1.00 – 2.00] |
| Fluvoxamine | N06AB08 | 107 | 1.00 [0.50 – 1.00] |
| Escitalopram | N06AB10 | 30 | 1.00 [1.00 – 1.00] |
| Oxitriptan | N06AX01 | 0 | - |
| Mianserin | N06AX03 | 0 | - |
| Trazodone | N06AX05 | 4 | 0.33 [0.33 – 0.33] |
| Nefazodone | N06AX06 | 0 | - |
| Mirtazapine | N06AX11 | 39 | 0.50 [0.50 – 1.00] |
| Bupropion | N06AX12 | 9 | 0.50 [0.50 – 0.50] |
| Venlafaxine | N06AX16 | 49 | 0.75 [0.73 – 0.75] |
| Duloxetine | N06AX21 | 4 | 0.75 [0.50 – 1.00] |
| Agomelatine | N06AX22 | 3 | 1.00 [1.00 – 1.00] |
| St. John’s wort | N06AX25 | 0 | - |
| **Total** |  | **1549** | **1.00 [0.50 – 1.00]** |

Supplemental table 3: Median [IQR] of the maintenance dose for all included antidepressants. Note: no defined daily dose has been set for St. John’s wort (N06AX25).
